# First human case of tick-borne encephalitis virus infection acquired in the Netherlands, July 2016

**DOI:** 10.2807/1560-7917.ES.2016.21.33.30318

**Published:** 2016-08-18

**Authors:** Joris A de Graaf, Johan H J Reimerink, G Paul Voorn, Elisabeth A bij de Vaate, Ankje de Vries, Barry Rockx, Alie Schuitemaker, Vishal Hira

**Affiliations:** 1 Department of Neurology, Zuwe Hofpoort Hospital, Woerden, The Netherlands; 2 National Institute for Public Health and the Environment (RIVM), Bilthoven, The Netherlands; 3 Department of Medical Microbiology, Zuwe Hofpoort Hospital, Woerden, The Netherlands; 4 Department of Medical Microbiology and Immunology, St. Antonius Hospital, Nieuwegein, The Netherlands; 5 Department of Internal Medicine, Zuwe Hofpoort Hospital, Woerden, The Netherlands

**Keywords:** tick-borne diseases, emerging diseases, Flaviviridae, public health, viral encephalitis, viral infections

## Abstract

In July 2016, the first autochthonous case of tick-borne encephalitis was diagnosed in the Netherlands, five days after a report that tick-borne encephalitis virus (TBEV) had been found in Dutch ticks. A person in their 60s without recent travel history suffered from neurological symptoms after a tick bite. TBEV serology was positive and the tick was positive in TBEV qRT-PCR. TBEV infection should be considered in patients with compatible symptoms in the Netherlands.

Until recently, tick-borne encephalitis virus (TBEV) was thought to be absent in the Netherlands and all cases of tick-borne encephalitis (TBE) were considered imported from endemic regions [1,2]. On 30 June 2016, the Dutch National Institute for Public Health and the Environment (RIVM) reported that Dutch *Ixodes ricinus* ticks were RT-PCR positive for TBEV-Eu, but no autochthonous cases had been diagnosed at that point [3]. This is the first report of an autochthonous case of TBE in the Netherlands.

## Case description

In June 2016, a person in their 60s presented at a hospital in the middle of the Netherlands with complaints of malaise, fatigue, headache, nausea and a subfebrile temperature (37.9 °C) after a tick bite. The malaise and fatigue had started earlier that month (day 0), the other symptoms started two days later. On day 4, the general practitioner discovered a tick on the patient’s left leg, removed it and started antibiotic treatment with doxycycline for 10 days. In retrospect, the bite is most likely to have occurred two days before onset of symptoms in a forested area between Driebergen en Maarn. Initially, the patient improved after antibiotic treatment and the symptoms disappeared. However, on day 12 the patient suffered from tremors, slow speech, weakness and fatigue. Subsequently, these symptoms progressed and fever (40.0 °C), nausea and vomiting developed on day 21. The patient was referred to the hospital on day 24. Neurological and general physical examination revealed no other abnormalities, especially no signs of meningism. Laboratory blood tests showed no specific abnormalities (Table 1), nor did a computed tomography scan of the brain. Serum and cerebrospinal fluid (CSF) tested negative for Lyme borreliosis (Table 2), however, CSF showed a mononuclear cell reaction (Table 1).

**Table 1:** Clinical, chemical and haematological tests on blood and cerebrospinal fluid at hospital admission, tick-borne encephalitis case, the Netherlands, July 2016*

	Patient	Reference value
**Blood**		
Haemoglobin (mmol/L)	7.6	8.5–11.0
Leukocyte count (10^9^/L)	6.9	4.0–10.0
Thrombocyte count (10^9^/L)	211	150–400
Erythrocyte sedimentation rate (mm/hour)	60	0–19
Sodium (mmol/L)	131	135–145
Potassium (mmol/L)	3.9	3.6–5.1
Glucose (mmol/L)	6.1	4.0–7.0
C-reactive protein (mg/L)	12	0–10
**Cerebrospinal fluid**		
Polynuclear cells (cells/µL)	2	Not applicable
Mononuclear cells (cells /µL)	61	Not applicable
Erythrocytes (cells /µL)	128	Not applicable
Glucose (mmol/L)	3.5	2.0–4.0
Protein (g/L)	0.89	0.15–0.45

**Table 2:** Performed tests for infectious diseases, tick-borne encephalitis case, the Netherlands, July 2016*

	Blood	Cerebrospinal fluid
*Bartonella henselae*	IgM negative	ND
*Borrelia burgdorferi*	C6 IgG negative IgG negative IgM negative	PCR negative serum/liquor index IgM and IgG negative
*Treponema pallidum*	Serological screening negative	ND
Tuberculosis	IGRA negative	ND
HIV	Serological screening negative	ND
Enterovirus	ND	PCR negative
Parechovirus	ND	PCR negative
Herpes simplex virus 1 and 2	ND	PCR negative
Varicella zoster virus	ND	PCR negative

HIV: human immunodificiency virus; IGRA: interferon gamma release assay; ND: Not done; PCR: polymerase chain reaction.

Additional diagnostic tests were conducted to exclude other infectious diseases (Table 2). Although TBE was not considered endemic in the Netherlands, it was added to the differential diagnosis after the RIVM reported that TBEV had been detected in ticks in the eastern part of the country (Sallandse Heuvelrug), 100 km from Driebergen [3]. In 2016, the patient had not travelled to the Sallandse Heuvelrug or any other regions known to be endemic for TBE. Their last stay abroad had been in October 2015, in Paderborn, Germany, which is not a region endemic for TBEV [4]. They had not visited other places abroad in the past five years. The patient was not vaccinated against TBEV, but had received a vaccination against yellow fever virus in 2005.

Serum taken on day 24 and 36 was positive for anti-TBEV IgM (452 and 162 Vienna units (VIEU)/mL, respectively; cut-off: 63 VIEU/mL) and IgG (> 650 and > 650 VIEU/mL, respectively; cut-off: 100 VIEU/mL) (Progen Biotechnik). CSF was negative for IgM but IgG-positive. In addition, both sera were positive in a TBEV neutralisation assay (1/640). A TBEV-specific qRT-PCR on CSF, blood and urine was negative. 

Fortunately, the patient had saved the dead tick, which was was positive for TBEV by qRT-PCR with a Ct value of 21. Interestingly, based on comparison of partial NS5 sequences of the PCR products, TBEV in the patient’s tick showed 93% homology with those found in Sallandse Heuvelrug, but 99% homology with a prototype TBEV-Eu Neudörfl strain.

During clinical observation, the patient gradually improved. At discharge on day 37, no focal neurological deficits were present, but fatigue and mild subjective cognitive complaints (Montreal Cognitive Assessment 26/30) remained.

## Discussion

This is the first report of a case of TBE in a patient infected in the Netherlands. Although liquor was negative for anti-TBEV IgM antibodies, the high serum IgM and IgG levels in an unvaccinated patient, combined with a typical biphasic clinical presentation and TBEV detected in the tick collected from the patient, confirmed the diagnosis of TBE [5]. Since the patient had not travelled abroad in the previous seven months, they must have been infected in the Netherlands, as the incubation period for TBE is no longer than a month and *Ixodes* species only feed for several days per host [1,6].

TBE is considered an emerging disease due to its rising incidence and the expansion in new, previously uninfected, areas but until now, autochthonous human TBEV infection had not been reported in the Netherlands [3,7]. The presence of TBEV in ticks collected in the Netherlands was recently confirmed [3]. Interestingly, preliminary sequence data suggest that the TBEV detected in the tick from our patient had a higher homology to the prototype TBEV-Eu strain Neudörfl than to those found in the Sallandse Heuvelrug. The Neudörlf strain and related TBEV strains have been found throughout Europe, including Germany. Although it is highly likely that the TBEV-infected tick that bit our patient was acquired between Driebergen and Maarn, the exact origin of the tick requires further investigation. Further studies are needed to determine the geographic spread and genetic diversity of TBEV in ticks in the Netherlands.

This case is an excellent example of the importance of tenacity and persistence in difficult diagnostic cases. Looking beyond guidelines and current evidence can lead to new findings, which can be beneficial not only for the individual patient but also for public health. Surveillance and widespread messages by public health institutes can be of great value to the diagnostic process, as they can provide clinicians with clues for the diagnosis of disease in individual patients. 

This case has important implications. On a patient level, clinicians in the Netherlands need to add TBE to the differential diagnosis for patients hospitalised with (meningo)encephalitis or meningitis who may have been exposed to tick bites. On a public health level, further studies are needed to determine the extent of TBEV infections in humans in the Netherlands. These studies include surveillance of TBEV in humans, animals and ticks, as well as determining the risk of acquiring TBEV infection by serosurveillance studies in the general population, patient populations with unknown neurological disease and for professions at high risk for tick bites.

## *Erratum

This article was originally published with an incorrectly ordered reference list. This was corrected on 19 August 2016. We apologise for this mistake.

On request of the authors, the date in the Table titles was corrected from June 2016 to July 2016. This change was made on 19 August 2016.
