# Erratum for Euro Surveill. 2016;21(33)

**DOI:** 10.2807/1560-7917.ES.2016.21.34.30323

**Published:** 2016-08-25

**Authors:** Eurosurveillance editorial team

**Affiliations:** 1 European Centre for Disease Prevention and Control, Stockholm, Sweden

In the article entitled ‘First human case of tick-borne encephalitis virus infection acquired in the Netherlands, July 2016’ by de Graaf et al. was published on 18 August 2016 with an incorrectly ordered reference list. This was corrected on 19 August 2016. We apologise for this mistake. 

In addition, on request of the authors, the date in the Table titles was corrected from June 2016 to July 2016. This change was made on 19 August 2016.

